# Beta-Lactamase-Producing *Escherichia coli* Isolates Recovered from Pig Handlers in Retail Shops and Abattoirs in Selected Localities in Southern Nigeria: Implications for Public Health

**DOI:** 10.3390/antibiotics10010009

**Published:** 2020-12-24

**Authors:** Olivia Sochi Egbule, Benson C. Iweriebor, Edward Ikenna Odum

**Affiliations:** 1 Department of Microbiology, Faculty of Science, Delta State University, Abraka 330106, Nigeria; odumedward07@gmail.com; 2 Department of Biology, School of Science and Technology, Sefako Makgatho Health Sciences University, Ga-Rankuwa, Pretoria 0204, South Africa; benvida2004@yahoo.com

**Keywords:** *Escherichia coli*, pigs, retail shops, abattoirs, extended-spectrum beta-lactamase-producing *Escherichia coli*, metallo beta-lactamase-producing *Escherichia coli*

## Abstract

Antibiotic resistance evolution among pathogenic microorganisms has become a huge burden globally as it has increased the burden of diseases amongst humans and animals. The prevalence of extended-spectrum beta-lactamase-producing *Escherichia coli* (ESBL-Ec) and metallo beta-lactamase-producing *Escherichia coli* (MBL-Ec) isolated from pig abattoir and handlers in retail shops was studied. In addition, the relationship between the isolates’ prevalence and the background characteristics of the butchers/retailers was also investigated. Samples from 32 hand swabs of pork sellers at retail shops and 8 butchers at abattoirs, as well as 272 swabs taken from knives, tables, floors, water troughs, and carcasses from both retail shops and abattoirs, were collected. *Escherichia coli* (*E. coli*) was isolated from hand swabs, fomites, and carcasses and were identified by standard microbiological procedures. The isolates susceptibility to nitrofurantoin (300 µg), ciprofloxacin (5 µg), ceftazidime (30 µg), cefuroxime (30 µg), gentamicin (10 µg), cefixime (5 µg), ofloxacin (5 µg), amoxicillin/clavulanic acid (30 µg), imipenem (10 µg), and meropenem (10 µg) and their ability to produce ESBL and MBL was determined by phenotypic methods. Demographic information of the handlers was retrieved by means of a structured questionnaire and, in some cases, via face to face interviews. Out of 104 *E. coli* isolates from both sources, 52 (50.0%) and 8 (7.7%) were ESBL and MBL producers, respectively. ESBL was more prevalent on the hands of the retailers (40.6%) and butchers (75.0%). The isolates were 100% resistant to ceftazidime, cefotaxime, and amoxicillin–clavulanic acid and 4.8% resistant to nitrofurantoin. Diverse resistance patterns were observed among ESBL-Ec and MBL-Ec. It was found that 90% of ESBL-Ec and 100% of MBL-Ec were multidrug-resistant. A possible epidemiological link between the two sources was observed. The prevalence of *E. coli* ESBL- and MBL-producing isolates was associated with the duty performed by handlers (*p* = 0.012) and gender (*p* = 0.012). Our results provide evidence that the handlers’ hands and abattoir environment had a great role to play in the high prevalence and resistance profiles of the microorganisms.

## 1. Introduction

*Escherichia coli* is an important Gram-negative bacillus (GNB) associated with several clinical and community infections [1]. The emergence of extended-spectrum beta-lactamase (ESBL)-producing bacteria, in response to the selective pressure of extensive cephalosporin use in the community, is of particular concern. ESBL-producing bacteria were initially observed in human clinical practice, but, in recent years, they have been increasingly recognized in food-producing animals and represent a growing problem involving food safety, with environmental implications [2,3]. Some reports have shown that food-producing animals are potential sources or reservoirs of ESBLs, which contribute to their spread in the community [4,5]. Increasing resistance of ESBL-producing bacteria to multiple types of antibiotics has led to an increase in the use of carbapenems [6], which, in response, has resulted in the emergence of carbapenemase-producing bacteria [7,8]. Carbapenemase enzymes produced by bacteria have the ability to hydrolyze all beta-lactams and are, therefore, of greater concern as far as management of infectious diseases is concerned. Over the past few years, carbapenem resistance due to metallo beta-lactamase (MBL) production has been increasingly reported among clinical isolates, and the ability to progress into the community as has already been demonstrated by extended-spectrum β-lactamase (ESBL)-producing GNB [9,10].

The issue of food safety is much more complicated in most developing countries of Africa; there are many informal markets, and a large percentage of the food produced is sold there, making enforcement of food safety regulations impracticable and often evaded. In Nigeria, the level of hygiene and food safety practices among meat handlers in retail shops and abattoirs is poor. Most rural and suburban regions lack essential amenities and food safety awareness [11]. Sanitary measures are often compromised, resulting in food contamination and the spread of pathogens harboring antimicrobial resistance determinants such as ESBL. The increasing distribution of ESBL-producing bacteria in the community contribute substantially to the community burden of antimicrobial resistance [12].

The true prevalence of extended-spectrum beta-lactamase-producing *Escherichia coli* (ESBL-Ec) from food animals is not known in Nigeria due to limited information. Even though the prevalence of ESBL-Ec has been reported in chicken and beef in Nigeria [13,14] in urban locations, information on pig retail shops and abattoirs in different parts of Nigeria is rare. However, a few studies on antimicrobial-resistant *E. coli* in pigs have been reported in Ibadan [15] and Lagos [16]. Though pork is highly consumed in Delta State, Nigeria, in a variety of local cuisines, there is, however, a paucity of epidemiological data on beta-lactam-producing *E. coli*, a common cause of community-acquired infection that is often associated with both pork production and consumption. To the best of our knowledge, no study on the occurrence of ESBL-Ec in pork carcasses, handlers, and abattoir environments has been undertaken in a rural Nigerian setting. The objective of this study, therefore, is to determine the prevalence of ESBL- and MBL-producing strains of *E. coli,* their antibiotic resistance patterns and the relationship between the sociodemographic characteristics of handlers in abattoirs and retail shops in the study communities.

## 2. Results

### Bacteria Identification and Phenotypic Resistance Profile

**Figure 1:**
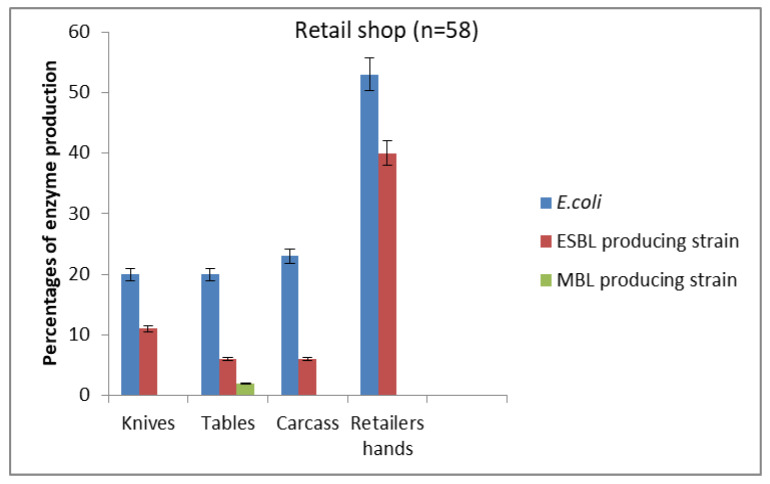
Prevalence of *E. coli*, extended-spectrum beta-lactamase (ESBL), and metallo beta-lactamase (MBL) producers isolated from retail shops.

**Figure 2:**
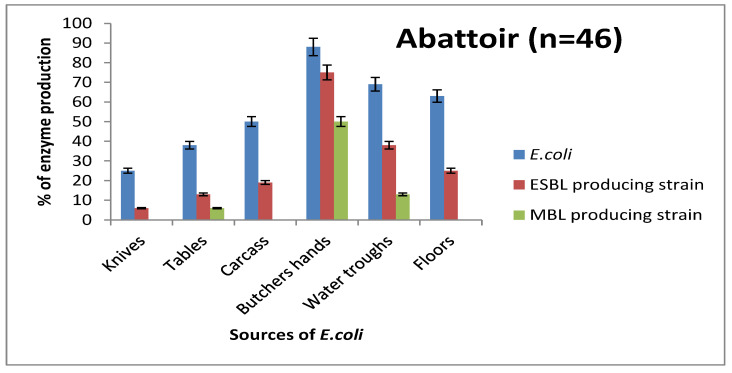
Prevalence of *E. coli*, ESBL, and MBL producers isolated from pig abattoirs.

The total number of *E. coli* isolated in this study was 104, out of which 58 and 46 were obtained from all sources tested in retailers’ shops (Figure 1) and abattoirs (Figure 2), respectively. Compared to other sources, *E. coli* was most prevalent in the hands of retailers (53.1%) and butchers (87.5%). Following the retailers’ hands in terms of the prevalence of *E. coli* at the retailers’ shops were carcasses (23.4%), then tables and knives (20.3%). At the abattoir, the highest prevalence was found among the butchers with 88 (88%), followed by water troughs with a prevalence of 69 (68.8%), floors 63 (62.5%), carcasses 50 (50.0%), tables 38 (37.5%), and knives 25 (25.0%). Though lower, the prevalence of ESBL producers followed the same trend as total *E. coli* (Figure 1 and Figure 2). The MBL producers were the least prevalent and, unlike ESBL producers that were found in all sources in retailers’ shops and abattoirs, MBL producers occurred at three sources, which were butchers’ hands, water troughs, and tables, in descending order, amongst the six sources in abattoirs and retail shops (Figure 1 and Figure 2). It is pertinent to note that all MBL-producing *E. coli* isolates coproduced ESBL, as can be seen in Figure 1 and Figure 2.

All the *E. coli* isolates were resistant to three antibiotics (ceftazidime, cefotaxime, and amoxicillin/clavulanic acid), while less than 5% of the isolates were resistant to nitrofurantoin (4.8%) (Table 1). Resistance to carbapenem (meropenem and imipenem) was very low, as 90% of the isolates were sensitive to this class of antibiotic.

In addition, resistance to both quinolone and cephalosporins were observed in 9 of the 14 resistance patterns exhibited by the isolates. Generally, resistance to other antibiotics was high (>58%) except for MER and IMP, which were marginally less than 10% (Table 1). The resistance patterns of ESBL- and MBL-producing *E. coli* isolates were similar about 71% of the time, regardless of the source (Table 2). In general, 14 diverse resistance patterns were observed amongst the 59 (ESBL and MBL) isolates; the spectrum of antibiotics to which ESBL and MBL were resistant to is shown in Table 2. Multidrug resistance (MDR-resistance to three or more classes of antibiotics) was observed in 90% (47/52) of ESBL isolates. The MDR ESBL isolates were resistant to at least two cephalosporins in addition to other drugs. The MBL isolates, although fewer in number, were all MDR.

The prevalence of the isolates varied with the background characteristics but was only significantly associated with duty and gender (Table 3).

Protocols for sanitation, hygiene, and prevention of meat contamination, which should include regular hand washing, disinfection of hands, utensils, and floors, use of clean protective clothes, and protection of displayed meat from flies, were completely absent except for disinfection and use of refrigerators/coolers; this was practiced by less than 30% of the butchers and retailers, as shown in Table 4. These portent risk factors for the spread and high prevalence of *E. coli* and ESBL producers were observed among the pig handlers in the rural locations of Delta state, Nigeria. Moreover, physical handling of money without due regard to basic hygiene is a common practice amongst all retailers and butchers (Table 4).

## 3. Discussion

The emergence of extended-spectrum beta-lactamase (ESBL)-producing bacteria, especially among members of the *Enterobacteriace*, has become of great importance in recent years as it has increased the global disease burden. Extended-spectrum-β-lactamase (ESBL)-producing organisms are increasingly prevalent worldwide and pose a serious public threat due to their assaults on most of the members of the cephalosporin group of antibiotics, which are the most effective weapons in the armamentarium against bacterial scourges on mankind. The incidence of infection with ESBL-Ec has been observed in food animals globally [8,17]; hence, its occurrence in pork meat and the related environments in this Nigerian setting does not come as a surprise. The finding of higher prevalence of ESBL on the hands of retailers and butchers can be attributed to poor hygiene practices, which education did not influence, as the results of this study show (Table 3). Thus, the findings substantiate the report of Founou and co-workers [18] with respect to the prevalence of bacteria on the hands of retailers and butchers and poor hygiene practice, especially when handling money during sales [19,20,21]. Further evidence of poor hygiene practice has come from the observation that butchers and retailers are associated with the prevalence of ESBL or MBL. The absence of training in food safety, which could have reduced the incidences of poor hygiene, as indicated by Nik et al. [22] and Lynch et al. [23], further compound the problem.

The high prevalence of ESBL-Ec isolated from the hands of handlers is an indication that, just as with nosocomial infections, hands constitute an important vehicle for the transfer of ESBL-producing pathogens in the livestock industry. Infection control measures on the transmission of ESBL-producing pathogens exist within hospitals but not for outside sources like veterinary settings. 

When compared to retail shops, abattoirs have a greater prevalence of *E. coli* isolates. This may be attributed to the poor environmental conditions and slaughtering operations, leading to the cross-contamination of carcasses. Several research papers on Nigeria have reported a similarly high prevalence of *E. coli* due to contamination of meat and the poor environmental conditions of slaughterhouses [24,25], showing that unsafe meat is being released for consumption in most parts of the country. It has been reported that disinfection or hand washing is poorly practiced, especially with water troughs, and the use of disinfectants or soap is rare in abattoirs. It is known that constant contact with live pigs is a factor for pathogen transmission [6,26] and the job title [5] increases the chances of colonization and transmission. Thus, frequent exposure to livestock may enhance the prevalence and dissemination of ESBL-Ec in humans when hygiene practice is lacking [27,28].

Of significance is that all isolates were resistant to cephalosporins, ceftazidime, and cefotaxime, which are antibiotics considered critically important by WHO [29]. These antimicrobial agents are widely used in Nigeria, thereby creating selective pressure and resulting in high resistance rates. The coexistence of the ESBL phenotype and quinolone resistance further supports previous studies that showed coproduction of beta-lactamase- and plasmid-mediated quinolone genes [8]. This contributes to frequent incidences of MDR and, thus, the high mortality rate associated with the resistance of ESBL-producing isolates to multiple antibiotics [30,31]. The low resistance of isolates to carbapenems, imipenem, and meropenem may be the reason for the low prevalence of MBL-producing *E. coli.* Carbapenems are considered a better option for the treatment of ESBL-Ec in pigs. However, a higher prevalence of 10% has been reported in Eastern Nigeria [32], which suggests a trend of rising prevalence of MBL-producing *Escherichia coli* in Nigeria.

The production of beta-lactamases is often of plasmid origin; hence, MDR is expected. A high prevalence of 90% MDR ESBL-Ec isolates was observed in this study (Table 2). Dissemination of MDR ESBL genes may have occurred because of the high prevalence of ESBL observed on handlers’ hands, poor sanitation, and hygiene measures. A study done in Thailand among layer chickens and pig farms reported MDR in all ESBL *E. coli* isolates [33]. Similarly, all ESBL *E. coli* isolated from pig feces by Nuanmuang et al. [34] were MDR.

It was observed that a similar phenotypic resistance pattern in the two broad sources of samples (abattoirs and retail shops) occurred, thereby indicating a possible epidemiologic link that presents a major risk to pig production and human health. Since pork is highly consumed in these selected rural communities of Delta State, Nigeria, there is, therefore, a greater risk of transmission of ESBL-producing *E. coli* from pigs to humans through the food chain. In addition, ESBL carriage has been associated with frequent consumption of pork [35]. There is, therefore, the need for constant risk assessment to reduce the occurrence and spread of this pathogen. Implementing measures to monitor and control the spread of these bacteria within veterinary settings is thus important.

## 4. Materials and Methods

### 4.1. Study Plan

This study was carried out between March and April 2019 in some rural communities of Delta State, Nigeria. These rural areas of Delta state were selected because of their relatively high consumption of pork. Pig production in the area is on the increase, and there is a high dependence on pork as a source of animal protein. A multistage sampling technique was adopted for the selection of locations and respondents. The primary stage involved the selection of two local government areas (LGAs), Ukwani and Ethiope-East, from the three agricultural zones in Delta State. Thereafter, 2 abattoirs and 16 retail shops per LGA were randomly selected for the second stage. Informed consent of the handlers was obtained before information on their background characteristics and swab samples were taken.

### 4.2. Sample Collection and Identification of E. coli

Swab samples from the palm and fingertips of 32 pork retailers and 8 abattoir butchers, as well as from cutting knives, tables, carcasses, abattoirs floors, and pigs’ water troughs, were collected using the swab/rinse method. Delineated areas of 10 × 10 cm were swabbed with sterile cotton swabs moistened with 1 mL sterile normal saline. The samples were collected once weekly for 2 weeks and transported in iceboxes to the laboratory and either processed within 2 h of collection or stored at 4 °C for subsequent analyses. 

The swab samples collected were homogenized by shaking manually with sterile normal saline and plated onto eosin methylene blue (EMB) agar petri dishes and incubated aerobically at 37 °C for 24 h. A single colony of *Escherichia coli*, characterized by green metallic sheen on EMB, was selected and subcultured onto nutrient agar plates for purification. Presumptive *E. coli* isolates were identified by biochemical tests, as described by [36].

### 4.3. Phenotypic Screening of Antibiotic-Resistant ESBL-Ec and MBL-Ec

Antibiotic sensitivity testing of all isolates was determined using the Kirby–Bauer diffusion testing method on Mueller Hinton agar (Oxoid, Hamshire, England). The antibiotics tested were (in µg) nitrofurantoin, 300; ciprofloxacin, 5; ceftazidime, 30; cefuroxime, 30; gentamicin, 10; cefixime, 5; ofloxacin, 5; amoxicillin/clavulanic acid, 30; imipenem, 10; meropenem, 10 (Abtek, Ahmedabad, Gujarat India). *Escherichia coli* ATCC 25922 was used as negative control throughout the study. Interpretation of antimicrobial susceptibility test results followed the guidelines of the Clinical Laboratory Institute [37]. 

Phenotypic detection of ESBL and MBL was carried out by double-disk synergy tests (DDSTs). For ESBL detection, 1 µL of the bacterial suspension was inoculated onto a Mueller Hinton agar (MHA) plate, and the inoculum was spread evenly with a sterile swab. Plates were allowed to dry at room temperature, then cefotaxime (30 µg) and ceftazidime disc (30 µg) were applied 20 mm apart from the center of an amoxicillin/clavulanic acid (20 µg/10 µg) disc. Plates were incubated at 37 °C for 24 h. The test was considered positive when the zone of inhibition around ceftazidime and cefotaxime was expanded in the presence of clavulanic acid by ≥5 mm.

For MBL detection, the test isolates, adjusted to 0.5 McFarland standard using distilled water, were inoculated on MHA plates [38]. An imipenem disc (10 µg) was placed on the plate, and a blank filter paper disc was placed at a distance of 10 mm. Thereafter, 10 µL of a 0.5 M EDTA solution was added to the blank disc. Enhancement of the inhibition zone between the imipenem and the EDTA-containing disc was considered positive.

### 4.4. Demographic Characteristics, Experience, and Training

Information on the background of the handlers was obtained by a structured questionnaire and, in some cases, via a face-to-face interview. None of the handlers attended safety training. The sanitary and hygiene practices of the handlers at the abattoirs and retail shops were assessed by visual observation using the following variables: availability and use of disinfectants and soaps; regularity of disinfection and hand washing; handling of money with bare hands during sales or slaughter; protection of displayed meat from flies, availability of refrigerators or coolers for storage of meat before sales; the use of clean chopping boards. 

### 4.5. Data Analysis

The data was analyzed using SPSS version 22. The association between the prevalence of *Escherichia coli*, ESBL, or MBL producers and the sociodemographic characteristics of retailers/abattoir handlers was analyzed using chi-square statistics.

## 5. Conclusions

We conclude that ESBL is highly prevalent in abattoirs and retail shops and that the handler’s hands have a large role to play in the high prevalence and transmission of resistance profiles observed in ESBL-Ec isolates. Additionally, poor hygiene practice could play a significant role in the exacerbation of the observed prevalence and antibiotic resistance patterns. There are limited therapeutic options for infections arising from ESBL isolates. The limitations of the study are, however, the small sample size and the lack of molecular approaches, which would have given a better picture of the genetic determinants of the observed resistance.

## Figures and Tables

**Table 1:** Phenotypic resistance profile of *E. coli* isolates.

Antibiotics	Resistant Strains (*n* = 104)	
	*n*	%
Nitrofurantoin (NIT)	5	4.8
Meropenem (MER)	10	9.6
Imipenem (IMP)	10	9.6
Ciprofloxacin (CIP)	65	62.5
Ceftazidime (CAZ)	104	100.0
Cefixime (CXM)	70	67.3
Gentamicin (GEN)	68	65.4
Cefuroxime (CFM)	104	100.0
Ofloxacin (OFX)	58	55.8
Amoxicillin/clavulanic acid (AMC)	104	100.0

**Table 2:** Resistance profiles of *Escherichia coli* producing ESBL AND MBL.

Resistance Pattern	No of Antibiotics Resisted	Sources of Isolates		No of Beta-Lactamase-Producing *E. coli*	
		Abattoir	Retail Shop	ESBL	MBL
NIT CIP, IMP, MER, CAZ, CXM, GEN, CFM, OFX, AMC	10	+	+	2	2
CIP, IMP, MER, CAZ, CXM, GEN, CFM, OFX	9	+	+	4	2
CIP, IMP, MER, CAZ, CXM, GEN, CFM, AMC	8	+	-	2	1
CIP, CAZ, CXM, GEN, CFM, OFX, AMC	7	-	+	3	
NIT, CIP, CAZ, CXM, GEN, CFM, AMC	7	-	+	1	
NIT, CAZ, IMP, CXM, CFM, AMC	6	-	+	1	1
CIP, CAZ, CXM, GEN, CFM, AMC	6	+	+	5	
CIP, CAZ, CXM, CFM, OFX, AMC	6	+	+	4	
CIP, CAZ, GEN, CFM, OFX, AMC	6	+	+	8	
CAZ, CXM, CFM, OFX, AMC	5	+	+	8	
CAZ, CXM, GEN, CFM, AMC	5	+	+	4	
CIP, CAZ, GEN, CFM, AMC	5	+	+	4	1
CAZ, CXM, CFM, AMC	4	+	+	1	
CAZ, CFM, AMC	3	+	+	5	

**Table 3:** The association between the prevalence of *Escherichia coli*, ESBL, or MBL producers and the sociodemographic characteristics of retailers/abattoir handlers.

Variables	Prevalence of *E. coli* [*n* (%)]			x^2^ (*p*-Value)
	Total *E. coli*	ESBL Production	MBL Production	
**Duty**				
Butcher	7	6 (85.7)	4 (57.1)	6.29 (0.012)
*Retailer*	17	13 (76.5)	0 (0.0)	
**Gender**				
Male	7	6 (85.7)	4 (57.1)	6.29 (0.012)
Female	17	13 (76.5)	0 (0.0)	
**Age**				
≤20	0	0 (0.0)	0(0.0)	3.51 (0.17)
21–30	5	4 (80.0)	1 (25.0)	
31–40	10	6 (60.0)	3 (30.0)	
>40	9	9 (100)	0 (0.0)	
**Marital status**				
Single	4	2 (50.0)	0 (0.0)	1.02 (0.60)
Married	17	15 (88.2)	4 (23.5)	
Widowed	3	2 (66.7)	0 (0.0)	
**Education**				
None/primary	18	14 (77.8)	4 (28.6)	1.35 (0.25)
Secondary	1	0 (0.0)	0 (0.0)	
Tertiary	5	5 (100)	0 (0.0)	
**Job experience (years)**				
1–5	1	1 (100)	0 (0.0)	1.07 (0.78)
6–10	4	2 (50)	1 (25.0)	
11–15	16	13 (81.1)	2 (12.5)	
above 16	3	3 (100)	1(33.3)	

**Table 4:** Sanitary and hygienic protocols by visual observation.

Variables	Retailer (%)	Butcher (%)
Availability of disinfectants and soap	5 (15.6)	2 (25)
Regular hand washing	0 (0.0)	0 (0.0)
Regular disinfection of hands, utensils, and floors	0 (0.0)	0 (0.0)
Use of clean protective clothes	0 (0.0)	0 (0.0)
Handling of money during sales or slaughter	32 (100)	8 (100)
Protection of displayed meat from flies	0 (0.0)	0 (0.0)
Availability of refrigerators or coolers	6 (18.3)	0 (0.0)
Use of separate chopping boards (flat tables) for cutting meat and intestinal parts	0 (0.0)	0 (0.0)

## Data Availability

All data presented in this study are available in article.
